# Terminal deoxynucleotidyl transferase‐positive high‐grade B‐cell lymphoma with *MYC* and *BCL2* rearrangements transformed from follicular lymphoma

**DOI:** 10.1002/jha2.1060

**Published:** 2024-12-02

**Authors:** Radu Chiriac, Lucile Baseggio, Marie Donzel

**Affiliations:** ^1^ Hematology Laboratory Hospices Civils de Lyon Centre Hospitalier Lyon Sud Lyon France; ^2^ Department of Pathology Hospices Civils de Lyon Centre Hospitalier Lyon Sud Lyon France

**Keywords:** follicular lymphoma, HGBCL, TdT

1

A 48‐year‐old man with a 2‐year history of classical follicular lymphoma (according to the 5th WHO classification) [1] involving the axillary lymph nodes achieved complete remission after six cycles of obinutuzumab plus cyclophosphamide, doxorubicin, vincristine, and prednisone. By the end of the third cycle of maintenance therapy with obinutuzumab, the patient presented with epigastric and left hypochondrial pain while in complete remission for 8 months.

Laboratory studies revealed a lactate dehydrogenase level of 2000 U/L (reference range: 135–235 U/L) and mild anemia (90 g/L). However, the peripheral blood smear showed 10% atypical intermediate‐sized lymphomatous cells, characterized by nuclei with oval to irregular contours, finely stippled chromatin, variable nucleoli, and intensely basophilic cytoplasm with numerous vacuoles (Figure 1A). Staging bone marrow was negative. Peripheral blood flow cytometry showed a kappa‐restricted population of mature B‐cells that were CD45+, CD19+, CD10+, CD5‐, and CD20‐ (Figure 1B). To evaluate the abdominal pain, ^18^F‐fluorodeoxyglucose (^18^F‐FDG) positron emission tomography/computed tomography revealed a left retroperitoneal solid mass with increased ^18^F‐FDG uptake, an SUVmax of 20, and measuring 6 × 16 cm (Figure 1C).

**FIGURE 1:**
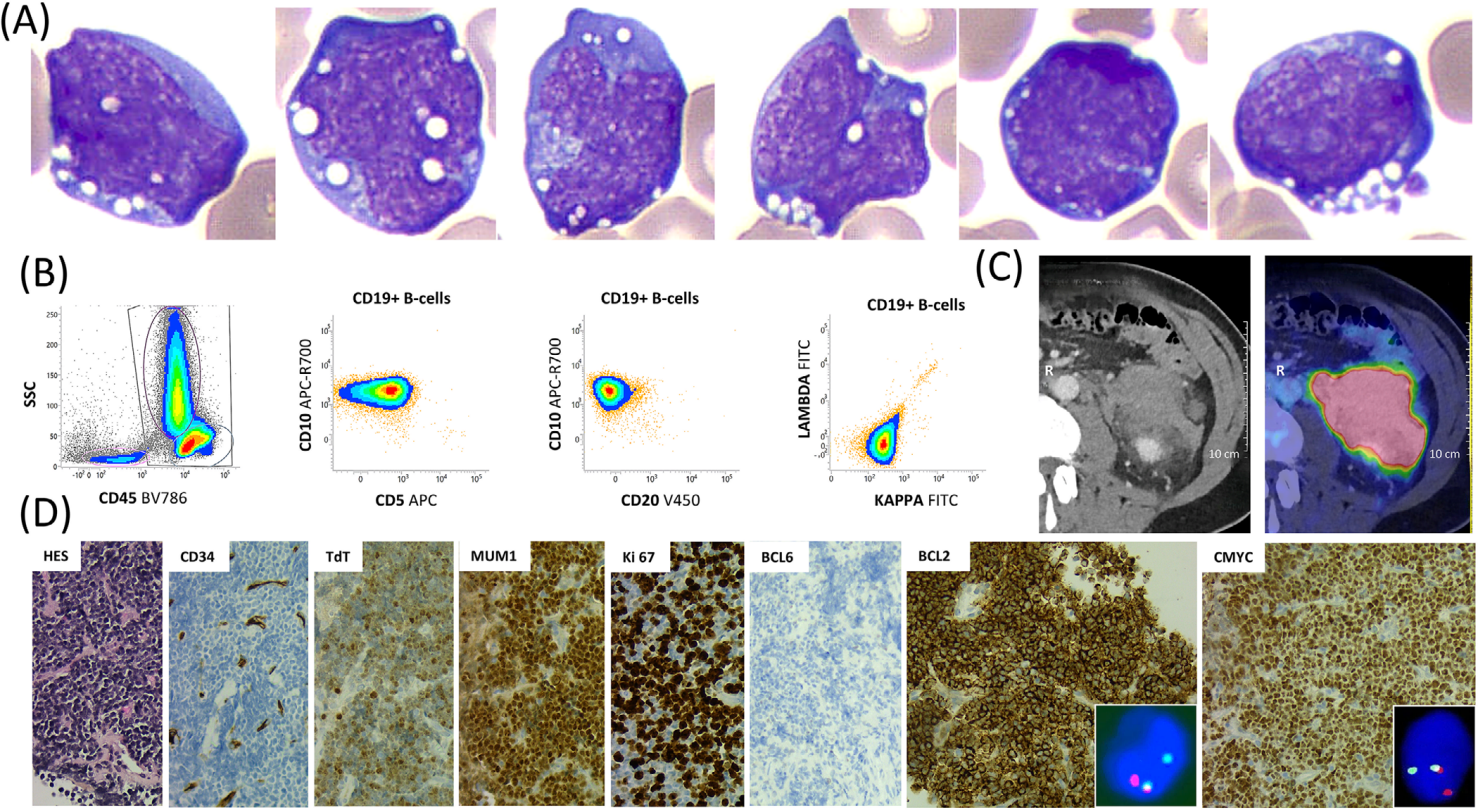
Panel (A) May‐Grunwald Giemsa stain, ×100 objective, showing intermediate‐sized lymphomatous cells with irregular nuclei, stippled chromatin, variable nucleoli, and basophilic cytoplasm with vacuoles. Panel (B) Flow cytometry results showing kappa‐restricted mature B‐cells that were CD45^+^, CD19^+^, CD10^+^, CD5^−^, and CD20^−^. Panel (C) ^18^F‐fluorodeoxyglucose (^18^F‐FDG) positron emission tomography/computed tomography (PET/CT) showing a left retroperitoneal solid mass with increased FDG uptake, SUVmax 20, measuring 6 ×16 cm. Panel (D) ×20 objective, Mass biopsy showing monomorphic medium‐sized cells (Hematoxylin Eosin Saffron stain) with CD34^−^, TdT^+^, MUM1^+^, high Ki‐67 index, BCL6^−^, BCL2^+^, c‐MYC^+^, *MYC*, and *BCL2* rearrangements (insets).

Mass biopsy revealed a predominance of monomorphic medium‐sized blastoid cells with scant basophilic cytoplasm, round nuclei, and conspicuous nucleoli. Mitotic figures were easily observed (Figure 1D). The neoplastic cells were positive for B‐cell markers, including PAX5 and CD19, but negative for CD20. They exhibited a germinal center phenotype (CD10+, BCL6‐, and MUM1+) and overexpressed both c‐MYC and BCL2. Diffuse terminal deoxynucleotidyl transferase (TdT) expression and monotypic surface immunoglobulin light chain expression were also observed. CD5, CD34, P53, and Epstein–Barr virus‐encoded RNA in situ hybridization were negative. The Ki‐67 proliferation index was 70%. Fluorescence in‐situ hybridization performed with break‐apart probes on tissue samples showed *MYC* (80%) and *BCL2* (90%) rearrangements, with no *BCL6* rearrangement (Figure 1D, insets).

Ifosfamide and etoposide‐based chemotherapy were initiated, followed by anti‐CD19 chimeric antigen receptor T‐cell infusion, which was initially well‐tolerated; however, a recurrence developed 3 months later. He was switched to dexamethasone, high‐dose cytarabine, and oxaliplatin but developed tumor lysis syndrome with renal failure despite prophylaxis. He experienced progressive disease involving the kidney, lower retroperitoneum, extraperitoneal space, and testis, ultimately dying 7 months after diagnosis.

This case describes a complex example of an aggressive TdT‐positive high‐grade B‐cell lymphoma (HGBCL), with *MYC* and *BCL2* rearrangements transformed from follicular lymphoma marked by significant tissue involvement and a concurrent leukemic phase. TdT‐positive HGBCL is a rare and aggressive mature BCL recognized in the 5th WHO classification [1]. It is important to differentiate between mature and immature B‐cell neoplasms, as they require distinct treatment strategies. B‐cell neoplasms expressing TdT without definitive features of immaturity may present a diagnostic challenge. In contrast to B‐lymphoblastic lymphoma/leukemia, TdT‐positive HGBCL typically exhibits a mature B‐cell immunophenotype, characterized by surface immunoglobulin light chain restriction, negative CD34, and strong positivity for CD38 and CD45. Furthermore, attention should be given to rare cases of B‐lymphoblastic leukemia/lymphoma that exhibit surface light chain expression [2]. This case demonstrates that TdT may be expressed rarely in mature BCLs, typically occurring at the time of disease progression or relapse.

## AUTHOR CONTRIBUTIONS

Radu Chiriac and Marie Donzel wrote the manuscript and conducted the cytomorphological examinations. Lucile Baseggio did the immunological examinations. All authors contributed to the final manuscript.

## CONFLICT OF INTEREST STATEMENT

The authors declare no conflict of interest.

## FUNDING INFORMATION

The authors did not receive support from any organization for the submitted work.

## ETHICS STATEMENT

This manuscript respects the ethics policy of CHU Lyon for the treatment of human research participants.

## PATIENT CONSENT STATEMENT

No patient‐identifying data were used. The authors did not obtain written informed consent from the patient but the patient did not object to his data being used for research purposes (as required by the ethics policy of CHU Lyon).

## CLINICAL TRIAL REGISTRATION

The authors have confirmed clinical trial registration is not needed for this submission.

## Data Availability

Data sharing is not applicable to this article as no new data were created or analyzed in this study.
